# Increased Gastric IL-1β Concentration and Iron Deficiency Parameters in *H. pylori* Infected Children

**DOI:** 10.1371/journal.pone.0057420

**Published:** 2013-02-25

**Authors:** Dulciene Maria Magalhaes Queiroz, Andreia Maria Camargos Rocha, Fabricio Freire Melo, Gifone Aguiar Rocha, Kádima Nayara Teixeira, Simone Diniz Carvalho, Paulo Fernando Souto Bittencourt, Lucia Porto Fonseca Castro, Jean E. Crabtree

**Affiliations:** 1 Laboratory of Research in Bacteriology, Faculdade de Medicina, Universidade Federal de Minas Gerais, Belo Horizonte, Brazil; 2 Endoscopy Service, University Hospital, Universidade Federal de Minas Gerais, Belo Horizonte, Brazil; 3 Department of Pathology, Faculdade de Medicina, Universidade Federal de Minas Gerais, Belo Horizonte, Brazil; 4 Leeds Institute of Molecular Medicine, University of Leeds, Leeds, United Kingdom; Centro di Riferimento Oncologico, IRCCS National Cancer Institute, Italy

## Abstract

Association between *H. pylori* infection, iron deficiency and iron deficiency anaemia has been described, but the mechanisms involved have not been established. We hypothesized that in *H. pylori* infected children increased gastric concentrations of IL-1β and/or TNF-α, both potent inhibitors of gastric acid secretion that is essential for iron absorption, are predictors for low blood concentrations of ferritin and haemoglobin, markers of early depletion of iron stores and anaemia, respectively. We evaluated 125 children undergoing endoscopy to clarify the origin of gastrointestinal symptoms. Gastric specimens were obtained for *H. pylori* status and cytokine evaluation and blood samples for determination of iron deficiency/iron deficiency anaemia parameters and *IL1* cluster and *TNFA* polymorphisms that are associated with increased cytokine secretions. Higher IL-1β and TNF-α gastric concentrations were observed in *H. pylori*-positive (n = 47) than in -negative (n = 78) children. Multiple linear regression models revealed gastric IL-1β, but not TNF-α, as a significant predictor of low ferritin and haemoglobin concentrations; results were reproduced in young children in whom *IL1RN* polymorphic genotypes associated with higher gastric IL-1β expression and lower blood ferritin and haemoglobin concentrations. In conclusion, high gastric levels of IL-1β can be the link between *H. pylori* infection and iron deficiency/iron deficiency anaemia in childhood.

## Introduction

Anaemia is a major public health problem in developing countries and approximately half of all cases are due to iron deficiency (ID) [1], [2]. Iron deficiency anaemia (IDA) is the final stage in the spectrum of a persistent negative iron balance, being preceded by an iron-restricted erythropoiesis characterized by low iron stores. The greater demand for iron due to growth, expansion of red cell mass and menstrual blood loss in female adolescents favors the development of ID/IDA in childhood/adolescence [2]. Factors which contribute to the high frequency of ID/IDA in developing countries include poor iron intake, low dietary iron bioavailability and blood loss due to gastrointestinal parasitic infections [2], [3].

*Helicobacter pylori* colonizes the stomach of more than a half of the world’s population. Concomitant high prevalence of *H. pylori* infection and ID/IDA in some areas, particularly in developing countries, has led to the hypothesis that *H. pylori* may contribute to ID/IDA. Potential mechanisms proposed include increased blood loss due to *H. pylori*-induced gastric lesions [4], iron uptake by *H. pylori* [5], deficient iron absorption due to decreased gastric acidity [6], [7], that isessential for the reduction and solubilization of non-heme iron, and reduced gastric juice ascorbic acid concentrations [6].

The acute phase of *H. pylori* infection is accompanied by transient hypochlorydria of variable duration [8]–[10]. Similar perturbations in gastric acid secretion occur in animals following infection with gastric *Helicobacter* species [11] or *H. pylori* [12]. In the latter study, reversal of the hypochlorhydria induced by *H. pylori* infection in gerbils by treatment with recombinant IL-1 receptor antagonist implicated the *IL-1B* gene cluster in hypochlorhydric response to *H. pylori*. As *H. pylori* infection is mainly acquired in childhood [13], it is biologically plausible that infected children are at increased risk of developing ID/IDA as a consequence of hypochlorhydria.

Concentrations of IL-1β and TNF-α, potent inhibitors of gastric acid secretion [14], are increased in the gastric mucosa of *H. pylori* infected adults and children [15]–[17]. As both cytokines are capable of directly inhibiting gastric acid secretion by parietal cells, they might be one of the links between ID and *H. pylori* infection in childhood [13]. Furthermore, *H. pylori* infected adults with functional polymorphisms in the *IL1* gene cluster associated with over expression of IL-1β have increased hypochlorydria [17] that could, theoretically, interfere with intestinal iron absorption leading to ID.

We hypothesized that in *H. pylori* infected children without the known common causes of ID, increased gastric concentrations of IL-1β and/or TNF-α could be predictors for low blood concentrations of ferritin and haemoglobin, markers of early depletion of iron stores and anaemia, respectively.

## Patients and Methods

This study was approved by the Ethics Committee of the Universidade Federal de Minas Gerais, Belo Horizonte, Brazil and the National Ethics Committee on Research from the Health Ministry of Brazil. Signed informed consent to participate was obtained from the children (whenever possible) and adolescents and their parents.

### Patients

Between June 2007 and July 2010 125 children and adolescents (74 girls and 51 boys, mean age 11.1±2.9 years, range 4–16 years) undergoing gastrointestinal endoscopy to clarify the origin of symptoms related to the upper gastrointestinal tract were prospectively studied. All the patients were from Minas Gerais state, localized in the Southeast Brazil. To avoid confounding factors which can modify the gastritis classification and the diagnosis of *H. pylori* infection, or which independently can modify iron stores, rigorous exclusion criteria were used. Exclusions included children who had received antimicrobial drugs, anti-cholinergic and steroidal and non-steroidal anti-inflammatory agents for at least 30 days, or proton pump inhibitors for at least 15 days before endoscopy; children with peptic ulcer disease, coeliac disease and intestinal parasitic infections; children with gastrooesophageal varices, coagulation disorders, acquired or congenital immunosuppression, inflammatory diseases, renal failure, hematological disorders and neoplasias. Following endoscopy, additional exclusion criteria were previously undiagnosed coeliac disease or any histological non-specific duodenal inflammation in the absence of duodenal gastric metaplasia. From each female adolescent data was obtained on age of menarche, interval between the menses, duration and amount of monthly flow. Those with heavy menstrual blood loss were not included in the study. The menstrual cycle was considered normal when the interval between flows was 25–31 days and duration between 3 and 5 days [18].

Biopsy specimens were obtained from the antral and corpus gastric mucosa for evaluation of *H. pylori* status, histological parameters and cytokine concentrations. Duodenal biopsies were taken for histological analysis and the exclusion of coeliac disease and tropical enteropathy. In addition, stool samples were obtained for parasitology and *H. pylori* monoclonal stool antigen assay (HpSA Plus; Meridian Bioscience, Cincinnati, OH).

Blood samples were collected to determine the haemoglobin concentration, haematocrit value, *IL1B, IL1RN* and *TNFA* polymorphisms and serum ferritin concentration.

### *H. pylori* Status

*H. pylori* status was evaluated by culture, preformed urease test, carbolfuchsin-stained histological section, polymerase chain reaction (PCR) for *ure*A, monoclonal HpSA and ^13^C-urea breath test as previously described [19], [20]. Patients were considered *H. pylori*-positive when culture was positive or at least two of the other tests were positive and *H. pylori*-negative when the results of all tests were negative.

### Histology

Endoscopic biopsy specimens were fixed in 10% formalin and embedded in paraffin wax, and 4-µm-thick histological sections were stained with haematoxylin and eosin for histological analysis according to the revised Sydney System [21] and with carbolfuchsin for detection of spiraled organisms. Mononuclear (MN) and polymorphonuclear (PMN) cell infiltrations as well as intestinal metaplasia and atrophy were graded as absent (0), mild (1), moderate (2), or marked (3).

### Blood Haemoglobin, Haematocrit and Ferritin Values

Blood haemoglobin concentration and haematocrit values were determined by using an automated electronic counter, Sysmex XT 1800i (Sysmex Corporation, Kobe, Japan). The serum ferritin concentration was determined by a chemiluminescence method employing the ADVIA Centaur® Immunoassay CP System (Siemens Healthcare, Erlangen, Germany).

### Gastric Cytokine Levels

IL-1β and TNF-α gastric concentrations were evaluated separately in biopsies from the lesser curvature of the antrum and from the greater curvature of the corpus by ELISA and were expressed as picogram of cytokine per milligram of protein (pg/mg protein) as previously described [14].

### *IL1B-31T/C*, *IL1RN VNTR* and *TNFA*-307G/A Genotyping

As *IL1B/IL1RN* and *TNFA* polymorphisms might affect transcript levels of *IL1B* and *TNFA*, respectively, they were investigated in blood DNA extracted by the QIAamp DNA mini kit (QIAGEN, GmbH, Hilden, Germany). *IL1B-31T/C* genotypes were determined with Taqman double-stranded SNP ID: rs4986790 (Applied Biosystems, Foster City, CA). *IL1RN* penta-allelic variable number tandem repeats (VNTR) was genotyped according to Mansfield *et al* [22]. The alleles 1, 3, 4 and 5 were classified into long and the allele 2 into short categories [22]. The *TNFA*-307G/A genotypes were investigated by PCR-restriction length polymorphism (RFLP) [22].

### Statistical Analysis

Data were analysed with SPSS statistical software package version 17.0 (SPSS Inc., Chicago, IL). Hardy-Weinberg equilibrium of alleles at individual loci was tested by χ2-test with Yate’s correction or Fisher’s exact text. The Kolmogorov-Smirnov goodness-of-fit was used to assess the normality of the data. When significant departures from normality were detected, the data were log-transformed. The histopathological scores in binary variables were transformed by combining absent/mild and moderate/severe scores to compare histopathological data with IL-1β and TNF-α gastric concentrations. The degree of gastric chronic (mononuclear) and active (polymorphonuclear) in *H. pylori*-positive and -negative children was compared by the two-tailed Mann Whitney U test. Correlations were evaluated by the Pearsońs correlation (continuous normally distributed data) or Spearmańs correlation (scores). The comparisons between antral and corpus cytokine concentrations were undertaken by two-tailed paired Student’s t test or Wilcoxon test. The level of significance was set at p≤0.05. Multiple linear regression analyses (“enter option”) were used in order to quantify the simultaneous and mutually independent contribution, of selected relevant predictor candidates, e.g. IL-1β and TNF-α gastric concentrations, for low ferritin and haemoglobin blood concentration (dependent variables) while controlling for confounders such as gender and age. Variables with p values ≤0.20 in the univariate analyses were selected for the multivariate analyses. The optimum sample size, based on a significant level of 0.05 and a statistical power of 0.80 (type II error 0.02) is 125 cases, even when only a small effect size (f = 0.10) is expected.

## Results

Among the 125 children, 47 (37.6%) were *H. pylori*-positive (mean age 11.7±2.7 years; 29 girls) and 78 (62.4%) were *H. pylori*-negative (mean age 10.7±2.9 years, 45 girls).

### Gastric Cytokine Concentrations

**Figure 1:**
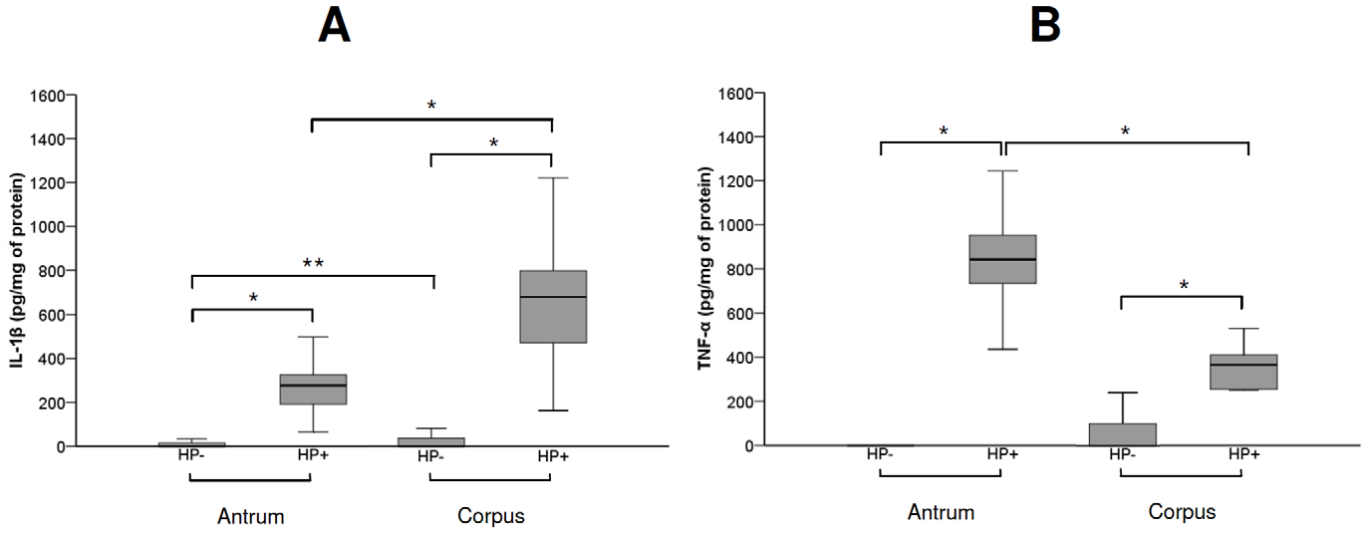
Box plots representing the comparison of gastric IL-1β (A) and TNF-α (B) concentrations (pg/mg of protein) between *H. pylori*-positive (HP+, n = 47) and -negative (HP-, n = 78) children, and between antral and corpus concentration in *H. pylori*-positive and -negative groups. The upper and lower limits of the boxes represent the 75^th^ and 25^th^ percentiles, respectively. The horizontal bar across the box indicates the median and the capped bars indicate the minimum and maximum data values. Statistical analysis by Student’s t test after log transformation in the case of IL-1β; *p<0.001 and **p = 0.02.

A 17.2-fold increase in corpus and antral gastric concentration of IL-1β as well as a 7.0-fold increase in corpus and 57.7-fold increase in antral gastric TNF-α concentrations were observed in infected children when compared with non-infected children (p<0.001). Comparisons between *H. pylori*-negative and -positive children are shown in Figure 1A and 1B. The concentrations of IL-1β were higher in the corpus than in the antral gastric mucosa (2.5-fold increase) in both *H. pylori*-positive (p<0.001) and *H. pylori*-negative (p = 0.02) children. Conversely, the TNF-α concentration was higher in the antral than in the corpus gastric mucosa in *H. pylori*-positive children (2.5-fold increase, p<0.001) (Figure 1A and 1B).

### Blood IDA Parameters and Gastric Cytokine Concentrations

The multiple regression analyses revealed the gastric concentration of IL-1β, but not TNF-α, as a significant independent predictor for low serum ferritin and haemoglobin concentrations (Table 1). In addition, the male gender was an independent predictor of ferritin increase and the age was an independent predictor of haemoglobin increase. As *H. pylori* infection is mainly acquired by young children and we have previously observed differences in the inflammatory and immune response between children younger and older than 10–12 years [23], we stratified children by age: 12 years old or younger, and those older than 12 years of age. IL-1β remained a predictor of low ferritin concentration and was a stronger predictor of low haemoglobin concentration in the younger age group. The male gender was an independent predictor of ferritin increasing (Table 1).

**Table 1:** Multiple linear regression model including ferritin or haemoglobin as dependent variables and IL-1β and TNF-α gastric corpus concentrations, gender and age as independent variables.

	Univariate analysis		Multivariate analysis	
	Betacoefficient	P value	Betacoefficient	P value
FERRITIN				
Children of all ages (n = 125)				
age	0.119	0.19	0.126	0.15
male gender	0.280	0.002	0.279	0.02
IL-1β	−0.200	0.006	−0.205	0.04
TNF-α	−0.075	0.41	–	–
Children ≤12 years of age (n = 84)				
age	−0.040	0.32	–	–
male gender	0.208	0.20	0.229	0.02
IL-1β	−0.202	0.03	−0.219	0.02
TNF-α	−0.106	0.35	–	–
HAEMOGLOBIN				
Children of all ages (n = 125)				
age	0.285	0.001	0.301	0.001
male gender	0.151	0.09	0.158	0.07
IL-1β	−0.220	0.02	−0.244	0.03
TNF-α	−0.135	0.14	−0.001	0.99
Children ≤12 years of age (n = 84)				
age	0.145	0.19	0.112	0.28
male gender	−0.170	0.12	−0.094	0.37
IL-1β	−0.399	<0.001	−0.439	<0.001
TNF-α	−0.167	0.13	0.092	0.49

**Figure 2:**
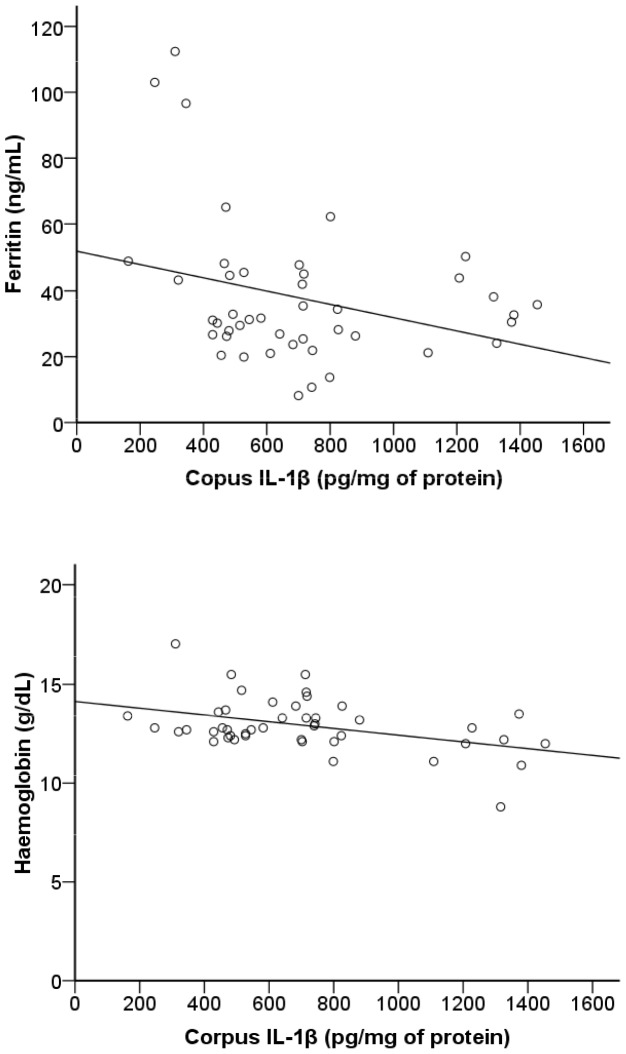
Correlations between corpus IL-1β concentrations and concentrations of ferritin and haemoglobin in *H. pylori*-positive children (n = 47). Statistical analysis by Pearson’s correlation after log transformation in the case of IL-1β and ferritin.

When *H. pylori*-positive and -negative children were separately analyzed, significant correlations were observed only in the *H. pylori*-positive group [ferritin (r = −0.42, p = 0.004) and haemoglobin (r = −0.35, p = 0.02)] (Figure 2). The values of haematocrit were also negatively correlated with gastric IL-1β concentrations (r = −0.40, p = 0.007]. In contrast, corpus and antral TNF-α concentrations neither correlated with serum ferritin (p≥0.48), nor with haemoglobin (p≥0.74) or haematocrit (p≥0.40) values.

### Gastric Inflammation and Cytokine Concentrations

The degrees of antral and corpus chronic and active inflammation were significantly higher (p<0.001 for all) in *H. pylori*-positive than in -negative children (Table 2). In *H. pylori*-positive children, the corpus concentration of IL-1β was significantly higher in children with a mild/moderate degree of corpus chronic inflammation than in those without corpus inflammation (672.48±300.37 pg/mg vs. 504.39±28.81 pg/mg, respectively, p = 0.002). Furthermore, the corpus concentration of TNF-α was higher (p<0.001) in infected children with a mild/moderate degree of corpus active inflammation than in those without corpus PMN cell infiltration (369.2±75.3 pg/mg vs. 277.8±75.3 pg/mg).

**Table 2:** Histological comparison of antral and corpus gastric mucosa of *H. pylori (HP)*-positive (n = 47) and -negative (n = 78) children^a^.

Inflammation	Absentn (%)	Mildn (%)	Moderaten (%)	Markedn (%)	P value
Antrum					
Chronic inflammation					
HP-positive	01 (2.2)	09 (19.5)	35 (76.1)	01 (2.2)	
HP-negative	34 (47.9)	37 (52.1)	0	0	<0.001
Active inflammation					
HP-positive	04 (8.7)	26 (56.5)	16 (34.8)	0	
HP-negative	66 (93.0)	5 (7.0)	0	0	<0.001
Corpus					
Chronic inflammation					
HP-positive	03 (6.8)	38 (86.4)	03 (6.8)	0	
HP-negative	42 (54.5)	35 (45.5)	0	0	<0.001
Active inflammation					
HP-positive	15 (34.1)	29 (65.9)	0	0	
HP-negative	74 (96.1)	03 (3.9)	0	0	<0.001

a One antral and 3 corpus gastric biopsy specimens from *HP*-positive and 7 antral and 1 corpus biopsy specimens from *HP*-negative children were deemed to be inadequate for histology assessment; n, number.

Neither atrophy, nor intestinal metaplasia was observed.

### *IL1B*, *IL1RN* and *TNFA* Polymorphisms

*IL1B* (p = 0.45), *IL1RN* (p = 0.36) and *TNFA* (p = 0.78) polymorphic genotypes did not associate with *H. pylori* status (Table 3). No association between *IL1B* and *TNFA* polymorphic alleles and blood ID/IDA parameters (p≥0.17) was observed.

**Table 3:** *IL1B*-31, *IL1RN* and *TNFA*-307 genotypic frequencies in *H. pylori-positive* (n = 47) and –*negative* children (n = 78).

	All childrenn (%)	*H. pylori*-positiven (%)	*H. pylori*-negativen (%)
*IL1B*-31^a^			
T/T	37 (29.8)	16 (34.8)	21 (26.9)
T/C	63 (50.8)	20 (43.5)	42 (55.2)
C/C	24 (19.4)	10 (21.7)	14 (17.9)
*IL1RN* VNTR^b^			
1/1	82 (66.2)	30 (65.2)	52 (66.7)
1/2	35 (28.2)	15 (32.6)	20 (25.6)
2/2	07 (5.6)	01 (2.2)	06 (7.7)
*TNFA*-307			
G/G	87 (69.6)	32 (68.1)	55 (70.5)
G/A	38 (30.4)	15 (31.9)	23 (29.5)

a It was not possible to genotype 1 *H. pylori*-positive children for *IL1B-*31 and 1 for *IL1RN*.

b 1 indicates all the long alleles and 2 the short allele. The loci did not deviate significantly from the expected Hardy-Weinberg distribution (P = 0.90 for *IL1B-3*1, P = 0.26 for *IL1RN* and P = 0.08 for *TNFA*-307) and all segregated independently.

In the *H. pylori*-positive group, carriers of the polymorphic *IL1RN* allele 2 had increased corpus inflammatory scores (p = 0.02) and significantly higher (p = 0.01 for both) IL-1β concentrations than the non-carriers (antrum 354.4±189.3 pg/mg vs. 253.5±74.3 pg/mg and corpus 873.4±466.6 pg/mg vs. 624.7±183.1 pg/mg). In the *H. pylori*-positive group of children of 12 years of age or younger (16 non-carriers vs. 9 carriers), a significant negative association between *IL1RN* polymorphic genotype and haemoglobin (13.0±0.9 g/dL vs. 11.9±1.3 g/dL, p = 0.03) or haematocrit (39.5±2.2% vs. 36.0±4.2%, p = 0.01) was observed.

The mean gastric concentration of antral (p>0.12) and corpus IL-1β (p>0.15) and antral (p>0.32) and corpus TNF-α (p>0.28) did not differ between carriers and non-carriers of *IL1B* and *TNFA* polymorphic genotypes, independently of the *H. pylori*-status.

## Discussion

ID is a very common micronutrient deficiency that affects individuals globally, but is a particular problem in developing countries. Children are considered to be at high-risk of ID that associates with deficits of immune, cognitive and motor functions [24], [25], in addition to the possible development of anaemia.

Accumulating evidence from epidemiological studies and clinical trials implicates *H. pylori* infection in the aetiology of ID/IDA [22], [26], [27]. Notably, ID in *H. pylori* infected subjects is resistant to iron supplementation, but this resistance is reversible by *H. pylori* eradication [28], [29]. However, the mechanism, or mechanisms, by which the infection might cause ID/IDA are still not determined. In the present study, we demonstrate that in *H. pylori*-infected children without common known causes of ID/IDA increased gastric IL-1β concentration is an independent predictor for low blood concentration of ferritin and haemoglobin.

Although it would be expected that in *H. pylori* infected children gastric IL-1β concentrations would be higher in the antrum, which is more inflamed than the corpus, a 2.5-fold increase in corpus IL-1β was observed. This could be explained either by increased corpus IL-1β production, or by a higher number of IL-1 receptors in the gastric corpus. In the former, as there was no corresponding increase in inflammation, IL-1β sources other than inflammatory cells have to be considered. However, taking in account that IL-1β inhibits gastric acid secretion by acting directly on parietal cells [14], [17], [30], the latter hypothesis seems more likely. Importantly, both murine gastric mucous and parietal cell lineages express the IL-1R1 receptor [31]. In contrast to IL-1β, the concentration of TNF-α was higher in the antrum than in the corpus, but TNF-α was not associated with blood iron parameters. However TNF-α concentrations were correlated with the scores of PMN cells in the corpus mucosa and inflammation has been considered a putative mechanism of disruption of the acid homeostasis.

The negative correlation between gastric IL-1β and ID/IDA blood parameters observed in this study could be due to the powerful capability of IL-1β in inhibiting gastric acid secretion [14]. Gastric acid is essential for iron absorption by reducing the ferric iron to a more soluble and absorbable ferrous iron form [2], [6]. The results of this study would support the argument that in the early phase of *H. pylori* infection, high gastric secretion of IL-1β that inhibits acid secretion impairs the absorption of iron. IL1β would also participate in the impairment of iron absorption by up-regulating hepcidin as demonstrated *in vivo* [31], [32]. However, in a recent study, Schwarz *et al* [33] did not observe associations between the serum concentrations of hepcidin and *H. pylori* infection.

In chronic *H. pylori* infection in adulthood, gastric atrophy leading to hypochlorhydria is considered a mechanism of iron deficiency. Gastric corpus atrophic changes are more frequently observed in adult carriers of the *IL1* gene cluster polymorphisms [17]. In the present study, *IL1RN,* but not *IL1B* polymorphism, was associated with increased gastric IL-1β concentration in concordance with studies in our adult population [34]. The latter study demonstrated that polymorphism of *IL1RN,* but not *IL1B,* is associated with increased risk of atrophic gastric changes and gastric carcinoma in this region of Brazil. In the current study we demonstrate that in the group of the *H. pylori*-positive youngest children, the haemoglobin and haematocrit values are lower in carriers of *IL1RN* polymorphic alleles than in children with the wild genotype. The high production of IL-1β in the former group might account for a more severe hypochlorhydria in the acute phase of *H. pylori* infection that is mainly acquired in early childhood.

In conclusion, we provide additional and convincing evidence of a role of gastric IL-1β induced by *H. pylori* infection in decreasing iron absorption in children. Thus, *H. pylori* infected children with ID/IDA may benefit from *H. pylori* eradication therapy.
